# Persistence and regredience of intraspinal fluid collection determine symptom control in intracranial hypotension syndrome

**DOI:** 10.1007/s10072-020-04609-w

**Published:** 2020-08-03

**Authors:** Gereon Johannes Schnellbächer, Michael Mull, Arno Reich

**Affiliations:** 1 Department of Neurology, RWTH Aachen University, Pauwelsstrasse 30, D-52074 Aachen, Germany; 2 Department of Diagnostic and Interventional Neuroradiology, RWTH Aachen University, Aachen, Germany; 3 Department of Neuroradiology, RWTH Aachen University, Pauwelsstrasse 30, D-52074 Aachen, Germany

**Keywords:** Intracranial hypotension, Headache, Intraspinal fluid collection, Superficial siderosis

## Abstract

**Background and purpose:**

An intraspinal fluid collection (ISFC) can be observed on spinal MRI in cases of intracranial hypotension syndrome (IHS). The goal of this study was to analyze the possible persistence of ISFC after therapy and its correlation to clinical disease activity and secondary complications.

**Materials and methods:**

Twenty patients in our database of 57 patients, who were treated for IHS between 2009 and 2015, fulfilled the inclusion criteria of (a) diagnosed and treated IHS as well as (b) an ISFC in MRI imaging. Ten of these participated in our study. We performed follow-up visits, which included a history, a clinical examination, and a spinal MRI.

**Results:**

A MRI-confirmed ISFC was seen in six patients, five of which had symptoms attributable to chronic IHS. There were two cases of superficial siderosis. One patient had a persisting ISFC and was free of symptoms. Four patients did not have an ISFC and were free of symptoms (Fisher’s exact test; *p* < 0.048).

**Conclusion:**

There is statistically significant correlation between the persistence of an ISFC after IHS treatment and ongoing clinical symptoms. Resolved symptoms seem to correlate with absorbed extradural ISFC and hypothetically closed leakage site. ISFC as confirmed by MRI proofs to be a reliable follow-up marker for disease activity in chronic IHS that is possibly even superior to clinical examination.

## Introduction

Intracranial hypotension syndrome (IHS) is a consequence of cerebrospinal fluid (CSF) hypovolemia, caused by spontaneous or (micro-)traumatic CSF leakage into extra-arachnoidal and extradural spinal spaces [1]. The estimated annual incidence is 5 per 100,000 with a midlife peak and a female predominance [2]. The diagnostic criteria are (A) any headache fulfilling criteria B through D with (B) low CSF pressure (< 60 mmH_2_0) and/or evidence of CSF leakage on imaging; (C) headache has developed in temporal relation to the low CSF pressure or CSF leakage or has led to its discovery (D) not better accounted for by another ICHD-3 diagnosis (Headache Classification Committee of the International Headache Society 2013). The overall spectrum of symptoms besides headaches is more diverse and commonly includes photophobia, dizziness, and tinnitus. Neurocognitive decline and brainstem ischemias have also been reported [3, 4].

In the case of chronic IHS, the headache may change from an initial postural character with severe headaches while standing into a milder and constant bilateral throbbing without clear orthostatic features [5]. This is important since it adds to the obscure nature of the disease and makes clinical observation more difficult. Although generally considered to be benign, especially chronic IHS may lead to secondary complications, such as cerebral sinus thrombosis and superficial siderosis (SS) [6], with potentially irreversible damage and in some cases even lethal consequences [7, 8]. These long-term sequels further expand the spectrum of symptoms that can be associated with an underlying chronic IHS pathology.

A variety of possible causes for the development of IHS exist including iatrogenic after surgery [9, 10]. In the past, a large proportion of IHS cases were considered to be idiopathic, since no clear etiology could be identified. In recent years, it has been suggested that microspurs and meningeal diverticula might explain many of those cases [11]. Independent of etiology spinal leakage leads to a common final path with CSF hypovolemia and extra-arachnoidal or extradural spinal fluid collection. First-line treatment is usually conservative, i.e., bedrest, head-down position, and administration of caffeine or theophylline, speculating on spontaneous closure of the leak over time. The next or alternative step is the application of repetitive blood patches without guidance. In cases of an identified leakage, CT-guided blood patches are performed [12, 13]. Moreover, an operative closure by administration of fibrin glue or a suture can be pursued.

The increased application of MRI frequently identifies not only supratentorial pathologies associated with IHS like subdural hematoma or the enhancement of the meninges but also an intraspinal fluid collection (ISFC) [14]. This fluid is hypothesized to be the accumulated leaked CSF. The exact anatomic compartment, in which spinal fluid collects, is sometimes difficult to assess and may vary from case to case. Both subdural and extradural extravasations of fluids have been described before [5]. In this study, the more general term ISFC is used to avoid confusion. In cases of chronic IHS with ISFC, the development of a SS is thought to be a serious complication. Hence, failure in leakage closure and ISFC resorption may lead to irreversible structural lesions of the central nervous system. The efficacy of the blood patches or surgery was often correlated to the reversal of cerebral abnormalities [15, 16]; however, more recently spinal alterations have become the focus of attention as well [17, 18]. The aim of the following study was to clarify the association of clinical disease activity with regard to persistence or regression of ISFC in chronic IHS and to study long-term sequels with regard to the occurrence of a cerebral hemosiderosis. We postulated that spinal MRI is a necessary and effective tool to monitor disease activity.

## Methods

### Patient selection

Adult patients who were treated at our university hospital between 2009 and 2015 were screened for eligibility, namely, the diagnosis of IHS and the presence of ISFC before treatment (Fig. 3). We used a search algorithm with the term “intracranial hypotension syndrome” for all patients treated in our institution in the above-mentioned time period. This resulted in 153 possible cases. After studying of the records, the diagnosis of IHS was confirmed in 57 patients. They had been dismissed from hospital care after resolution or reduction of the initial symptoms. Twenty-seven patients had suffered from a significant orthostatic headache after lumbar puncture, which could be treated without complications. The other 30 cases were diagnosed with an IHS of varying etiologies, such as spontaneous, after orthopedic infiltration therapy or traumatic. Of those 20 patients demonstrated a clear ISFC on MR imaging, 11 were willing to participate in the study and a written informed consent was obtained and 9 patients did not consent to participation. The study was approved by the local Ethics Committee and is in accordance with the Declaration of Helsinki.

### Examination and imaging

Since the occurrence of an ISFC had been described before [14], spinal MRI was routinely performed to screen for abnormalities. Additionally, postmyelographic CT imaging of the entire spine was obtained via lumbar puncture, and in most cases, the contrast medium was injected with the patient positioned in the CT table. Based on the area of interest, examination was started in a supine or prone position. In one patient, digital subtraction myelography was added, because CT myelography failed to show the CSF leak. The detailed techniques of CT myelography and digital subtraction myelography are described elsewhere [19–21]. Prospectively, we performed a follow-up visit consisting of a neurological examination with special focus on the history of headaches and signs of ongoing or newly acquired peripheral or central nerve symptoms. Symptoms were considered to be chronic, if they persisted for more than 3 months. Furthermore, a 3 Tesla MRI was used for the follow-up neuroradiological examination. Imaging protocol included sagittal imaging of the entire spinal column with T2-TSE, T1-TSE, T2* (MEDIC) sequences. The slice thickness was 3 mm. In addition, the region of the initial leak was examined axially with T2-TRUFI, T1-TSE; T2-TSE, T2* (MEDIC) sequences with a slice thickness of 3 mm.

### Statistical methods

The data was transferred on a fourfold table. A two-tailed Fisher’s exact test was used for statistical analysis. A *p* value of ≤ 0.05 was considered to be significant. Outcome variables were the existence or absence of ISFC in relation to symptoms typical for a chronic IHS. For the calculation, SPSS 23 was used.

## Results

Ten patients were examined and included in our data analysis. The eleventh patient underwent radiological follow-up examination, stated to be free of symptoms, but did not attend neurological follow-up examination. Since he did not comply with the study protocol, he had to be excluded. Of those participating in the study, four were female and six male (medium age 51.1 years). Median time from therapy to follow-up was 3 years (Q1 2 years, Q3 6 years).

### Clinical presentation

All showed as initial symptom of orthostatic headaches with one exception (case no. 4), where neck pain was the leading symptom. In this case, an IHS was diagnosed due to the presence of bihemispheric hygromas, ISFC, and identification of a thoracic dural leak. Four patients had additional focal neurological deficits (Table 1).

**Table 1:** Symptom constellations, neuroradiological results, and patient information.

Case #	1	2	3	4	5	6	7	8	9	10
*Symptoms at diagnosis*	Orthostatic headache, cranial nerve IV palsy	Orthostatic headache, muscle atrophy	Orthostatic headache	Neck pain	Orthostatic headache	Orthostatic headache	Orthostatic headache, cranial nerve III palsy	Orthostatic headache	Orthostatic headache, hypesthesia	Orthostatic headache
*Co-medication*	None	Antihypertensive	None	None	None	None	Antihypertensive	None	None	None
*Co-diseases*	None	Hypertension, prostatic hyperplasia	None	Deep venous thrombosis, fibromyalgia	Degenerative vertebra, mild brain atrophy	B12-deficiency	Nicotine abuse, hypertension	Disc prolapse	Migraine, lumbar skoliosis	Chronic heart failure, gastritis
*Imaging*	cCT sMRI myelography (CT)	cMRI sMRI myelography (CT) digital subtraction myelography	cMRI sMRI myelography (CT)	cCT cMRI sMRI myelography (CT)	cCT sMRI myelography (CT)	cMRI sMRI myelography (CT)	cCT cMRI sMRI myelography (CT)	cCT cMRI sMRI myelography (CT)	cCT sMRI myelography (CT)	cCT sMRI myelography (CT)
*Cerebral pathologies associated with IHS at diagnosis*	Bihemispheric hygroma	Unknown	Bihemispheric subdural hematoma	Bihemispheric subdural hematoma	None	Bihemispheric hygroma	Bihemispheric subdural hematoma	Bihemispheric subdural hematoma	N one	Bihemispheric subdural hematoma
*ISCF at diagnosis*	T1-T7 D	C6-T12 V	C3-C5 D C7-L2 V T9-T10 D	C2-T2 V C6-T11 D	T2 R L5 R	C7 V T2-T12 D	L5 V	C2-T3 V T1-T3 D T8-T12 D T10-L4 V	C4-T3 V	C2-T3 V
*Site(s) of leakage*	Not identified	T1/2 VM	T11/12 L L1/2 R	T6 R, VM T7 L T8 R	L4/5 R	T2/3 L	T8 R	T2/3 VM	T5 L T6 L T7 L	T1 VM
*Etiology*	Traumatic	Bone spike T2/3 R	Bone fragment T8/9 L	Spontaneous	Lumbar infiltration	Spontaneous	Spontaneous	Disc prolapse T2/3 M	Traumatic	Spontaneous
*Therapy*	Conservative	Surgery (2×)	Blood patch surgery	Blood patch	Blood patch	Conservative	Surgery	Surgery	Blood patch	Blood patch surgery
*Duration until follow-up (years)*	2	15	3	2	3	0.4	4	6	2	8
*Symptoms at follow-up*	Cranial nerve IV palsy	Chronic headache hypacusis muscle atrophy	Chronic headache concentration deficiencies	Neck pain	None	Chronic headache, speech problems	None	None	None	Gait ataxia hypacusis
*ISCF at follow-up*	absent	C7-T10 V T2-T3 V	C7-T6 V T10-L1 V T11 DL	T12 V	C6-T2 V T1-T9 D T9-L5 V	C4-T5 V T7-T12 V C7-T5 D	absent	absent	absent	C4-T1 V
*Long-term complications*	None	SS	subjective cognitive impairment	None	None	speech problems	None	None	None	SS

(cMRI, cerebral MRI; sMRI, spinal MRI; cCT, cerebral CT; V, ventral; DL, dorsolateral; VM, ventromedial; L, left; R, right; M, medial)

At the time of follow-up examinations, IHS-related headache symptoms were completely reversible in six patients. One patient described both a postural and non-postural headache. Two patients suffered from a chronic headache that did not show an orthostatic character. One patient reported a reduction of the initial neck pain. A paresis of the abducens nerve was persistent in one case after otherwise successful treatment possibly as a sign of irreversible nerve damage. Two cases developed over the course of up to 13 years a SS with ataxia and hypacusis at the time of follow-up. Furthermore, two patients complained about problems with concentration and verbal deficiency, respectively (Table 1).

### Neuroradiological presentation

Most of the leaks were found on the thoracic level (*n* = 8) at diagnosis. In one patient, the leak was located in the lumbar region. In one case, the site of the leakage could not be found. ISFC localization varied with four cases demonstrating extradural liquor ventrally, four cases both dorsally and ventrally, one case showed a right-sided ISFC, and one case only dorsally. Subdural hematomas or hygromas were present in seven cases.

At the time of follow-up examination ISFC could still be observed in six cases. Three of those had an ISFC ventral of the spinal cord and three demonstrated a fluid collection both ventrally and dorsally. From those cases still demonstrating an ISFC, in five several spinal levels were involved. In one case, ISFC could only be observed on level T12. Four patients demonstrated an increase of ISFC extent, while two patients showed a decrease. In four cases, the MRI demonstrated complete absorption the ISFC.

### Statistical analysis

Of ten patients, four had neither any symptoms nor a residual fluid collection at the time of follow-up. In one case, there was an absence of IHS while ISFC was still detectable. Five patients showed persisting symptoms and extradural fluid collections. There was no patient with ongoing symptoms while lacking a spinal fluid collection. The results were summarized in a fourfold table (Table 2) with statistical significance of *p = 0.04*.

**Table 2:** Fourfold table correlating chronic IHS symptoms to the persistence of ISFC at the time of follow-up

	Chronic IHS symptoms	No chronic IHS symptoms
Intraspinal fluid	5	1
No intraspinal fluid	0	4

## Discussion

Spinal imaging has become an essential part in the diagnosis and therapy of IHS [22]. Identification and localization of the CSF leak(s) may be difficult and influenced by the flow character in the leakage (low flow versus high flow). Different myelographic techniques (fast or ultra-fast CT myelography, digital subtraction myelography) were developed to overcome this problem [19, 20]. Spinal MRI can demonstrate various secondary spinal manifestations of IHS like ISFCs, dilated veins, and dural enhancement [2]. Supratentorial changes, as, for example, subdural hygromas, that are usually included into follow-up examination to ascertain regression are increasingly complemented by spinal imaging as well [18]. Since repeated or chronic microbleeds in IHS cases with an associated ISFC can lead to complications like SS [23] with potentially irreversible neurological deficits, spinal imaging rightfully deserves our attention. It has been theorized that drag caused by intracranial hypotension leads to an engorgement of intradural veins causing the subsequent microbleeds [24] and accumulation of hemosiderin on the brain surface and its subpial layers [25, 26] resulting in a cytotoxic effect through free iron and hydroxyl radicals [27]. Etiology of SS may vary with traumas, arteriovenous malformations, and tumors being among others [6]. However, in about half of the patients, no etiology is identified [28]. It is in these cases one has to be aware of the possibility of an underlying chronic IHS, which may be identified by the diagnosis of an ISFC. In cases #2 (Fig. 2) and #10, the leak persisted despite of the regression of orthostatic headache and led over a period of 15 and 8 years, respectively, to the development of a SS with debilitating symptoms like among others gait ataxia and hypacusis. Case #2 demonstrated SS of significant extent affecting frontal, sulcal, occipital, and intrathecal structures possibly due to the long time course disease progression was not stopped.

**Fig. 1:**
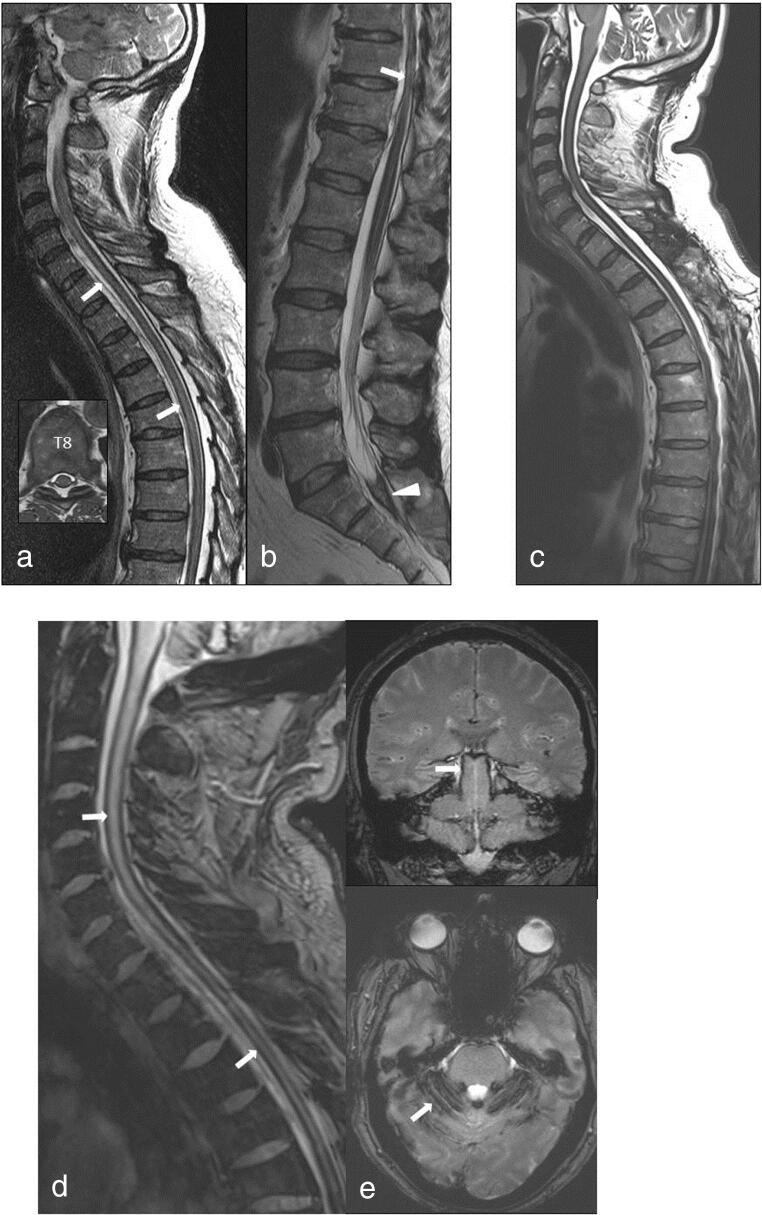
Preoperative sagittal and axial (inset) T2-weighted MR images of the cervicothoracic and lumbar regions (**a**, **b**) reveal extensive anterior intraspinal fluid collection from C5/6 to T 11 (arrows). Note hypointense formation of the distal thecal sac due to hemosiderin deposits at S1/2 (arrowhead). Postoperative sagittal T2-weighted MR images (**c**) 13 months after repair of the T1/2 dural tear shows persisting resolution of the fluid collection. Gradient echo MR images (**d**, **e**) clearly detect hemosiderin depositions along and around the cord associated with cord atrophy, as well as along the cerebellar folia, around midbrain and pons (arrows)

Not every leak seems to be accompanied by classical symptoms of IHS. Case #5 (Fig. 3), which originally demonstrated a recurrent leak at level L4/5 following facet infiltration, showed an extensive intraspinal fluid collection at the time of follow-up. However, the initial symptoms of IHS had ceased, and the patient presented himself free of complaints. A possible explanation is the development of a new equilibrium between the different spinal compartments and the surrounding connective tissue preventing further spreading while the leak itself remains unclosed. According to the Kelly-Monroe doctrine, the space within the central nervous system is limited. If one of the containing elements therein—blood, liquor, and brain parenchyma—increases in volume, the others must decrease. Through an extradural extension of liquor, this above-mentioned principle may be compensated and clinical symptoms may not be manifested. This may add to the variable and inconclusive clinical picture of chronic IHS and emphasizes the need for neuroradiological control examinations (Fig. 3).

**Fig. 2:**
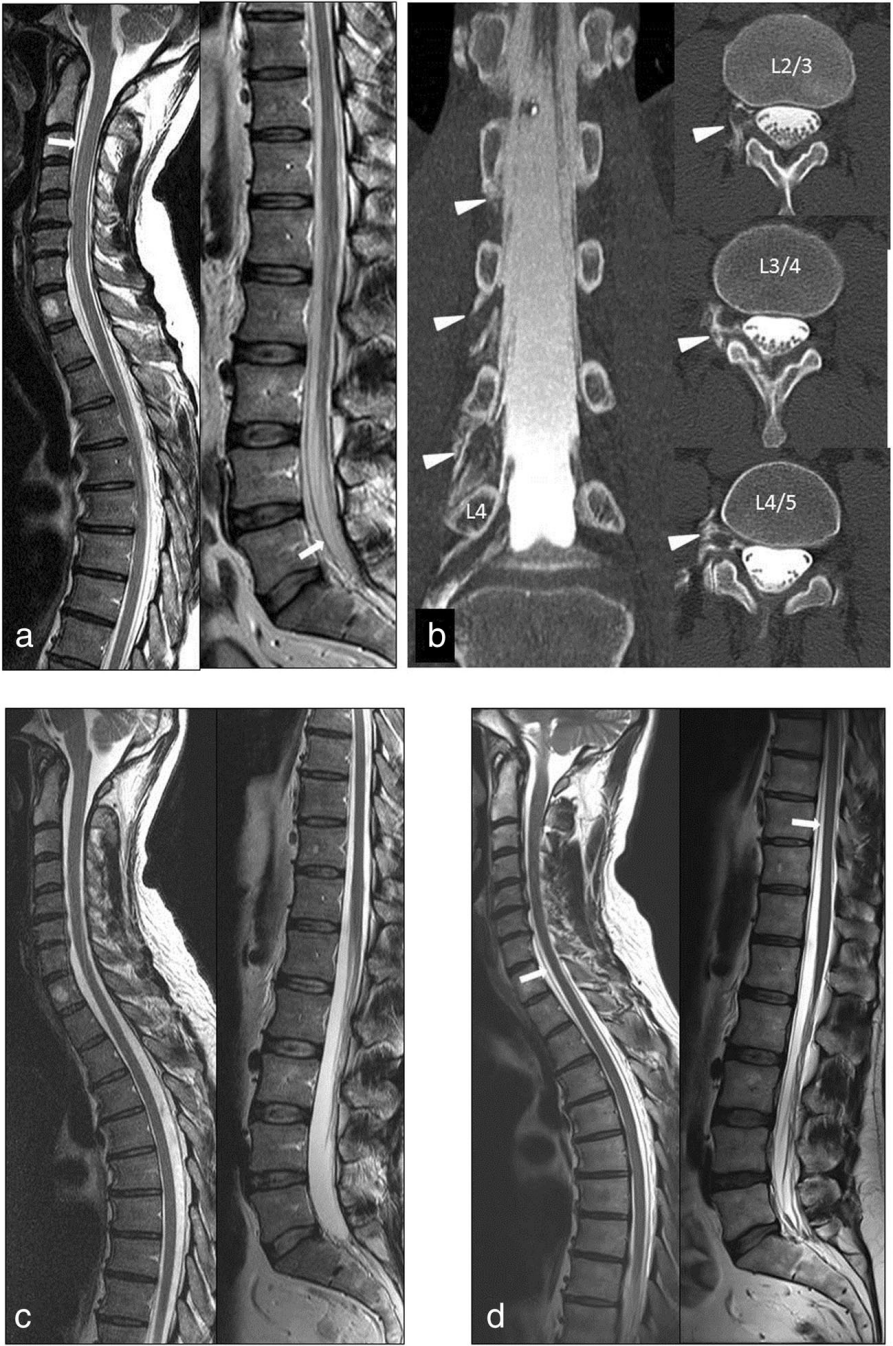
Initial sagittal T2-weighted MR images of the cervicothoracic and lumbar regions (**a**) demonstrate extensive anterior and posterior intraspinal fluid collections from C1 down to S1 (arrows). Coronal and axial reformatted CT myelography (**b**) reveals right-sided CSF leaks at the lumbar roots L2, L3, and L4 (arrowheads). Resolution of the intraspinal fluid collections is documented in sagittal T2-weighted MR images 1 month after a single lumbar CT-guided blood patch application (**c**). De novo formation of the intraspinal fluid collection (arrows) is observed without clinical symptoms in the late follow-up MR 3 years later (**d**)

**Fig. 3:**
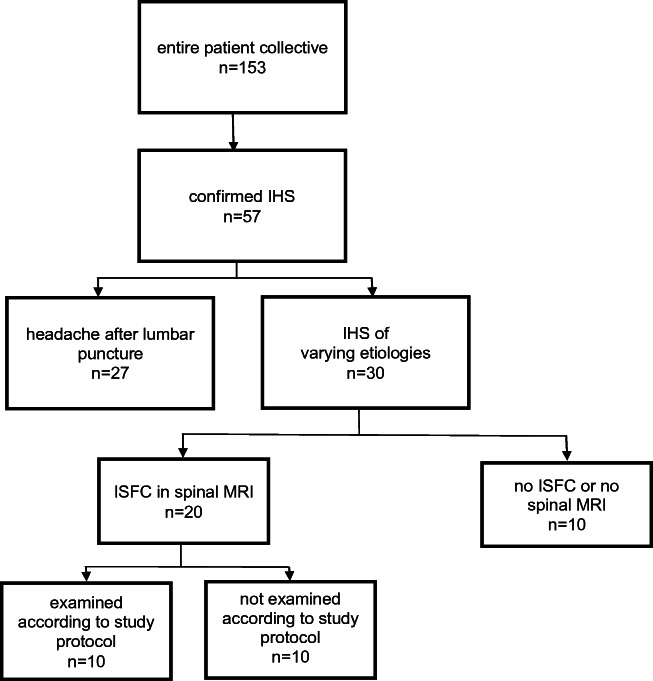
Flow-chart showing number of patients included in study

**Fig. 4:**
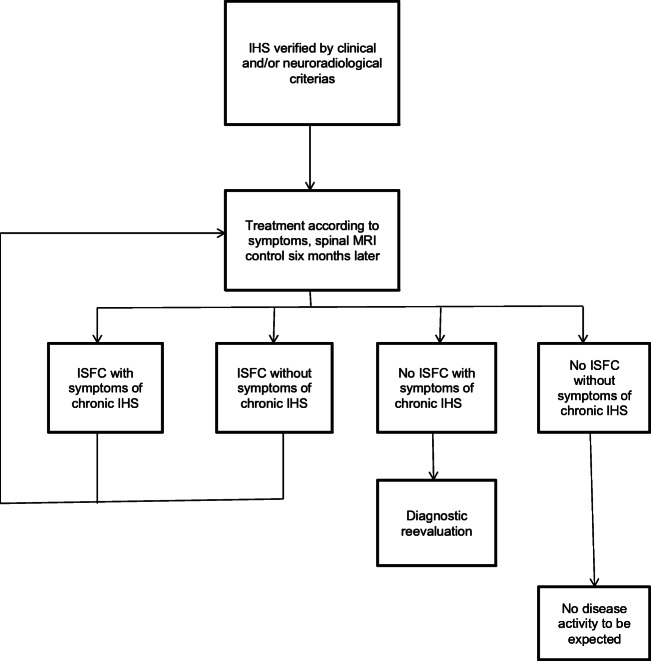
Decision tree for diagnostic evaluation

To our knowledge, this is the first study analyzing a long-term follow-up of IHS. In our cohort of patients, the median time interval from therapy to follow-up exam was 3 years. While this might be too short for reliably detecting SS, it can identify a recurrent or not regressing ISFC as described in case #5. It is of interest to note that both cases with SS had a particularly long time interval from therapy to follow-up. This indicates that complications of chronic IHS can manifest themselves even more than a decade after diagnosis. Figure 4 proposes a decision tree to be used in case of IHS. If an ISFC accompanies the diagnosis of an IHS, spinal follow-up examination is always recommended regardless of symptom control or regression. In our diagram, we propose an examination interval after therapy of 6 months to ascertain reduction of the ISFC. When no neuroradiological signs are present, the opaque nature of the clinical appearance of chronic IHS can make diagnosis difficult. In this case, a diagnostic reevaluation should take place in order not to miss possible treatable other causes for the complaints. In patients free of ISFC and without symptoms, no disease activity is to be expected.

The study was not designed to determine the incidence of an ISFC in patients with IHS. However, our retrospective analysis of patients with chronic IHS in our institution suggests that an ISFC can be found in a significant percentage of cases.

The protocol of spinal examinations should encompass T1 and T2 spin echo sequences for demonstration of intraspinal fluid collections. While the T2 spin echo sequence is effective in showing the dural sac, the T1 sequence is sensitive to the extradural fluid collection itself and useful to differentiate from epidural fat. A T2* or SWI sequence should be chosen to detect more sensitively possible cases of SS [29]. Another GRE sequence T2-Trufi can reduce the impact of field inhomogeneities caused by motion for an improved visualization of CSF.

Limitations of our study were its single-center design and the varying post-therapeutic follow-up intervals. The low number of patients recruited is problematic, although results were statistically highly significant. Further investigations could help improve both patient care and our understanding of a still insufficiently comprehended disease. Our data shows that persistence and dynamic of intraspinal fluid collections were predictive of chronic IHS-associated symptoms. All patients with a resolved fluid collection were free of symptoms on follow-up, while apart from one exception (case #5), all patients with persistent fluid collections suffered from ongoing complaints attributable to IHS disease. It can therefore be postulated that in case of successful closure of a leak, the intraspinal fluid is gradually absorbed and that the hypothesis of that spinal MRI is a valid tool for monitoring disease activity in IHS is true. It even seems more reliable than clinical examination since disease activity is often opaque and unspecific. It should routinely be used not only in the diagnosis of IHS but also in follow-up examinations. This is recommended not only for patients with ongoing chronic IHS symptoms but also for asymptomatic patients in order not to miss treatment opportunities and to prevent irreversible long term sequelae such as SS.
